# Parental overproduction allows siblicidal bird to adjust brood size to climate-driven prey variation

**DOI:** 10.1093/beheco/arae007

**Published:** 2024-02-01

**Authors:** Iván Bizberg-Barraza, Cristina Rodríguez, Hugh Drummond

**Affiliations:** Posgrado en Ciencias Biológicas, Universidad Nacional Autónoma de México, 04510 Ciudad de México, México; Departamento de Ecología Evolutiva, Instituto de Ecología, Universidad Nacional Autónoma de México, 04510 Ciudad de México, México; Departamento de Ecología Evolutiva, Instituto de Ecología, Universidad Nacional Autónoma de México, 04510 Ciudad de México, México; Departamento de Ecología Evolutiva, Instituto de Ecología, Universidad Nacional Autónoma de México, 04510 Ciudad de México, México

**Keywords:** facultative siblicide, insurance hypothesis, last-hatched offspring, neotropical seabird, overproduction, reproductive value, resource-tracking, sibling facilitation

## Abstract

Parental overproduction is hypothesized to hedge against uncertainty over food availability and stochastic death of offspring and to improve brood fitness. Understanding the evolution of overproduction requires quantifying its benefits to parents across a wide range of ecological conditions, which has rarely been done. Using a multiple hypotheses approach and 30 years of data, we evaluated the benefits of overproduction in the Blue-footed booby, a seabird that lays up to three eggs asynchronously, resulting in an aggressive brood hierarchy that facilitates the death of last-hatched chicks under low food abundance. Results support the resource-tracking hypothesis, as low prey abundance (estimated from sea surface temperature and chlorophyll-a concentration) led to rapid brood reduction. The insurance hypothesis was supported in broods of three, where last-hatched chicks’ survival increased after a sibling’s death. Conversely, in broods of two, results suggested that parents abandoned last-hatched chicks following first-hatched chicks’ deaths. No direct evidence supported the facilitation hypothesis: the presence of a last-hatched chick during development did not enhance its sibling’s fitness in the short or long term. The value of last-hatched offspring to parents, as “extra” or “insurance” varied with indices of food abundance, brood size, and parental age. Ninety percent of overproduction benefits came from enabling parents to capitalize on favorable conditions by fledging additional offspring. Our study provides insight into the forces driving overproduction, explaining the adaptiveness of this apparently wasteful behavior and allowing us to better predict how overproduction’s benefits might be modified by ocean warming.

## Teaser

Blue-footed Boobies’ production of excess chicks that often end-up dying appears wasteful but serves various purposes. Given the uncertainty over food availability, overproduction enables boobies to capitalize on favorable conditions by fledging additional offspring. Even in unfavorable conditions, extra chicks can benefit parents by replacing unexpectedly deceased siblings. This behavior helps boobies cope with reproductive uncertainties, but as ocean warming continues, overproduction’s benefits appear to diminish.

## INTRODUCTION

Production of offspring that exceed parents’ capacity or willingness to rear to independence is a widespread and important life-history trait with implications for fitness and demographics. Overproduction connotes increased competition among siblings for parentally provided food, which often leads to the selective death of the least competitive brood members (brood reduction). Typically, it is the last-hatched (“marginal”) individuals that die (Mock and Forbes 1995).

Several potential benefits of overproduction have been suggested. The resource-tracking hypothesis posits that overproduction of young hedges for unpredictability of food availability among breeding attempts, allowing parents to capitalize on favorable seasons. When resources are sufficient, marginal young are raised (extra reproductive value), when insufficient they die in the course of feeding competition (Lack 1947, 1954). The insurance hypothesis suggests that marginal young serve to replace others that succumb to illness, accidents, or predation before independence (insurance reproductive value, Dorward 1962). Finally, it is widely suspected that survival and fitness of the brood could be enhanced by marginal young through cooperation, exploitation, or developmental benefits of cohabitation, for example, through thermoregulatory benefit via huddling (Dunn 1976, 1979), by eliciting increased parental provision (Wilson and Clark 2002) or by facilitating the development of fighting skills and dominance (Bebbington et al. 2017).

Functions of parental overproduction are not mutually exclusive, yet only a few studies, mostly in temperate birds, have examined and quantified them under a multiple-hypothesis approach (Mock and Parker 1986; Wiebe 1996; Forbes et al. 2002; Humphries et al. 2006; Ferrer-Pereira et al. 2023). These documented both resource-tracking and insurance benefits of marginal young to adults’ reproductive success and showed that their magnitude and relative importance could vary across and within species depending on extrinsic and intrinsic conditions. Nevertheless, variation in the benefits of parental overproduction across environments and individual lifespans remains largely unexplored. The resource-tracking benefit is expected to be greater in birds with long developmental periods and high variability in food abundance, as resources available during the rearing period will be harder to anticipate (Forbes et al. 2001). Insurance benefit should be more important for birds with a high risk of partial brood failure due to predation or a high number of offspring per breeding event and in birds whose short breeding seasons limit the possibility of renesting (Forbes 1990). Tests of the facilitation hypothesis have focused on short-term benefits to siblings among temperate songbird nestlings (Wilson and Clark 2002), and the possibility of long-term developmental enhancement of siblings during adulthood has rarely been addressed (Forbes 2007). Not surprisingly, because tests need to control experimentally for heterogeneity in parental quality and follow individuals into adulthood. Finally, neotropical birds, which exhibit distinct life-history constraints (reviewed in Grémillet and Boulinier 2009; Svagelj 2019), have seldom been examined, limiting our ability to generalize.

We tested the three focal hypotheses using longitudinal data collected over 30 years on a marked and highly philopatric population of the Blue-footed booby. For each validated hypothesis, we quantified the contribution of last-hatched chicks to parents’ reproductive success and determined how it varied across environmental conditions and parents’ lifespans.

The Blue-footed booby is a long-lived neotropical marine bird that inhabits environments subject to the extreme ecological fluctuations of El Niño Southern Oscillation (ENSO, Ballance et al. 2006; Ancona et al. 2011) and the Pacific Decadal Oscillation (Fiedler 2002), and experiences prolonged and often severe brood-mate competition for food. Females lay 1–3 eggs of similar size and composition (Drummond et al. 2008), but most broods in the study colony are of one (≈47%) or two chicks (≈44%), and broods of three are uncommon (≈9%). Only 60% of two-chick broods and 42% of three-chick broods fledge all brood members (Bizberg-Barraza et al. unpublished data). Unreduced broods are most common early in the breeding season, as the frequency of brood reduction increases with laying date (Drummond et al. 2003). Hatching intervals (four days on average in broods of two and three) facilitate the formation of dominance relationships in broods of two and linear dominance hierarchies in broods of three (Valderrábano-Ibarra et al. 2007). First-hatched chicks’ daily aggression toward their siblings, which is sustained over the entire nestling period, starts at age 5–9 days and peaks in the third or fourth week. Notably, parents do not interfere in any obvious way in sibling competition (Drummond et al. 1991). Dominance ensures feeding priority and in the event of brood reduction determines which chick dies (Guerra and Drummond 1995). Under food shortage dominant chicks increase their aggression (Drummond and Garcia Chavelas 1989), in extremis to the point where subordinates starve or depart the nest in search of adoption, usually dying in the attempt when attacked by adult neighbors (Drummond et al. 1986). Experimentally lengthened or synchronous hatch intervals elicited greater aggression by dominant chicks and increased food provision by parents without affecting the probability of brood reduction, implying that natural intervals minimize the cost of parental care and increase the efficiency of brood reduction (Guerra and Drummond 1995). Booby offspring in the study population face predation during the egg and hatchling stages but maybe not during adult life. Sixty-one percent of eggs disappear from the nest, mostly predated by Heermann’s gulls (Mayani-Parás et al. 2015), and 16% of hatchlings are predated by (gape-limited) Atlantic Central American milk snakes (*Lampropeltis polyzona*) during the first four days of life (Ortega et al. 2021). These slow-moving nocturnal and crepuscular predators are the only known predators of chicks in the study colony. Blue-footed boobies appear to lack an effective defense against them; snakes enter their nests and ingest a single hatchling in the presence of parents or after dragging it away from the nest (Ortega et al. 2021).

We tested the resource-tracking hypothesis by evaluating whether the probability of brood reduction varies with two proxies of food availability: sea surface temperature (SST) and chlorophyll-a concentration (Chl-a) (Lanz et al. 2009). The nesting period in this species, from the start of incubation (which lasts ~ 40 days) to fledging at age 70 days, is long and marked by intra-annual fluctuations in prey fish abundance (Lanz et al. 2009). The hypothesis predicts that low food availability, associated with high SST or low Chl-a after laying, should increase the probability of brood reduction.

To test the insurance hypothesis, we analyzed the survival of last-hatched and first-hatched chicks following the death of a sibling. Under the hypothesis, a last-hatched chick’s survival should increase after the death of its sibling; in contrast, a first-hatched chick’s survival should be unaffected by its sibling’s death as its dominant status buffers against effects of competition (Forbes and Lamey 1996).

Finally, we evaluated the facilitation hypothesis by leveraging milk snake predation of hatchling chicks as a natural brood reduction experiment. Under the untested assumption that parents of depredated nests are similar in quality to parents of un-depredated nests, the hypothesis predicts that first-hatched chicks raised alongside their siblings should fledge with better body condition, be more likely to recruit into the breeding population, or have greater reproductive success during their first 6 years of life than first-hatched chicks whose siblings were depredated.

## METHODS

### Study species and data collection

We studied the highly philopatric (Kim et al. 2007) population of Blue-footed boobies on Isla Isabel, Nayarit, Mexico (21°50ʹ57ʹʹ N, 105°52’53” W). Most breeders recruit at 2 to 6 years, with males recruiting half a year on average after females (Oro et al. 2010). Boobies are socially monogamous, and both parents contribute similarly to incubation, brooding, and nest defense (Guerra and Drummond 1995). However, females are larger and provision chicks with three times as much food (anchovies, sardines, and herrings) as males (Guerra and Drummond 1995).

Every year, from 1989 to 2019, all nests in our study areas (2.6 ha) were monitored over the breeding season from February through July. Even though most females make only one breeding attempt each year, renesting can occur (6% of all nests), generally after nest failure. We included only females’ first broods of the season when testing the three hypotheses. During nest inspections, every 3–6 days, breeders were identified and sexed based on vocal dimorphism (females grunt, males whistle), and chicks were marked with colored plastic bands after hatching, then measured (ulna length, mm), weighed and fitted with numbered steel bands at 70 days, when they fledged (Drummond et al. 2003). Hatchlings were considered depredated when they disappeared (no cadaver was found in or near the nest) before reaching the estimated age of 7 days (details in Ortega et al. 2021). We set the upper age limit higher than the maximum age of hatchlings recovered from snakes’ stomachs to be sure of including all depredated hatchlings. When the cause of death is starvation, developmental failure, or siblicide, cadavers are commonly found on or near the nest.

To test for effects of feeding conditions, historical daily climatic and oceanic data were extracted from the National Oceanic and Atmospheric Administration (NOAA, http://www.nodc.noaa.gov) and averaged over a 900 km^2^ square centered on Isla Isabel (average foraging range of this species, Zavalaga et al. 2008; Weimerskirch et al. 2009). These data include sea surface temperatures (SST; AVHRR Pathfinder Version 5.3), rainfall (CHIRPS Version 2.0) from 1989 to 2019, and chlorophyll-a concentration (Chl-a; Chlorophyll-a, Aqua MODIS) from 2003 to 2019, an index of ocean productivity (Behrenfeld et al. 2006) which is closely correlated with the abundance of boobies’ prey (Lanz et al. 2009).

### Statistical analyses

#### General statistical procedures

All analyses were performed using R software 4.0.3 (R Core Team 2023). Model diagnostics for ordinary linear regression (from the “lme4” package, Bates et al. 2015) and general linear models (from the “glmmTMB” package, Brooks et al. 2017) were visually inspected using the “DHARMa” package (Hartig and Lohse 2021). Cox proportional-hazards model assumptions were verified using the “survminer” (Kassambara et al. 2021) and “survival” (Therneau 2020) packages. All covariates were standardized (centered and scaled), and collinearity was assessed using the variance inflation factor analysis from the “performance” package (Lüdecke et al. 2021). Time-dependent Cox models were employed to incorporate covariates that varied throughout the reproductive cycle, such as SST, Chl-a, and rainfall. To achieve this, the data were transformed into a counting process style (Andersen and Gill 1982). This means that each individual can have multiple rows corresponding to different time intervals, along with the time-varying covariate values specific to each interval (details in Zhang et al. 2018). Rainfall was included in all analyses as a control variable, as it can affect chicks’ thermoregulation and survival (Boersma and Rebstock 2014; Ortega et al. 2022). Prior to hypothesis testing, correlation tests confirmed that Chl-a and SST are adequate proxies of food availability (for details, see Supplementary Material): chick body mass at 70 days was negatively and positively related to SST and Chl-a, respectively (Supplementary Table S1a, b).

#### Resource-tracking hypothesis

To identify the relative time windows at weekly intervals (i.e., number of weeks prior to the event of interest of each individual) in which SST, Chl-a, and rainfall explained most of the variation in brood reduction probability, we implemented van de Pol et al.’s (2016) “sliding window” analysis using the R-package “climwin.” This not only identifies the best time window but also the function and aggregate statistics that best describe the relationship between environmental variables and the biological response. In the absence of prior evidence suggesting a linear relationship (Aranzamendi et al. 2019; Ortega et al. 2022), we tested both linear and quadratic functions, as well as multiple aggregate statistics (i.e., max, min; the mean was also tested for SST, and cumulative sum for Chl-a and rainfall). Due to convergence problems with rainfall and “min” as the descriptive metric, this combination was not included.

Preliminary analyses determined the optimal initial time window large enough to contain all environmental signals (time frames that explain the biological response), yet not so large as to reduce the probability of finding false positives (van de Pol et al. 2016). Randomization analyses were performed to assess the likelihood that a signal found was of biological relevance rather than occurring by chance. The best time windows of the relevant variables were refitted by re-running the sliding window analysis but this time incorporating in the model the best-fitting time windows for the remaining relevant variables to confirm the existence of multiple effects on the response variable. The windows we report were estimated by averaging the models that compose 95% of the total model weights (W_i_) (Bailey and van de Pol 2016).

To test whether high SST and low Chl-a after laying increased the probablity of brood reduction (brood reduction: at least one chick died = 1, neither chick died = 0), the sliding window analysis was carried out in broods of two from 1989 to 2019 in which no hatchlings were depredated (*n* = 2315 broods) because broods of three were too few to perform a meaningful sliding window analysis. Critical time windows for SST, Chl-a, and rainfall were identified using a Cox proportional hazard model from the package “survival” (Therneau 2020) as our “climwin” baseline model. Because brood hatching interval (days between hatching of first and last eggs of a clutch) and laying date (expressed as the relative rank between breeders of the same year; values close to zero indicate early breeders while values close to one indicate late breeders) affect intensity of sibling aggression (Osorno and Drummond 1995) and brood reduction (Drummond et al. 2003), respectively, those two terms were included in the model as covariates. The boundaries selected through preliminary analyses for the initial windows were 10 weeks prior to brood outcome (brood was reduced or both chicks fledged) for rainfall and 16 weeks prior to brood outcome for SST and Chl-a.

Once the best-supported environmental signals were identified and extracted, they were retested in a Cox model with the R-package “coxme’ (Therneau and Grambsch 2000), where parents’ ages (linear and quadratic terms) were added to the model to test their effects on brood reduction, along with year and nest ID as random effects to control for the sampling year and avoid pseudo-replication (*n* = 758 broods). The effects of mothers” and fathers’ ages were evaluated separately by alternating both terms in the model. This approach assures larger sample sizes and mitigates overrepresentation of longer active nests. Identifying both parents’ ages through their respective band IDs usually required the nest to be active for at least two consecutive nest inspections. The final model thus includes the most influential environmental variables, hatching asynchrony, laying date, and mother/father age as fixed effects.

#### Insurance hypothesis

The insurance hypothesis was examined by analyzing survival over the first 70 days of life of first- and last-hatched nestlings from broods of two (first-hatched chicks: *n* = 1948; last-hatched chicks: *n* = 1766) and three chicks (first-hatched chicks: *n* = 818; last-hatched chicks: *n* = 701) of birth cohorts 1989–2019. Two Cox models were fitted separately with the R-package “coxme,” to test if last-hatched offsprings’ survival increased following the death of first-hatched siblings in broods of two, and following the death of either elder sibling in broods of three. Another two Cox models tested if the survival of first-hatched chicks was affected by the death of last-hatched siblings in broods of two and the death of either younger sibling in broods of three. In all Cox models, survival (0) or death (1) of the focal chick was the response variable, and the survival (0 = none of its siblings died) or death (1 = one or more siblings died) of the focal chick’s sibling(s) prior to focal chick outcome (death/fledging) was added as a covariate of interest. Laying date, hatching interval, average SST, and rainfall experienced by the focal chick throughout the rearing period (from hatching to dying/fledging) were also included as control covariates, and year and focal chick ID as random effects. Inconsistencies in sample sizes between first- and last-hatched chicks arise from the exclusion of last-hatched individuals whose survival after fledging of their siblings was unknown because colony monitoring ceased before they reached 70 days old.

Chl-a data (available only from 2003 onwards) was not included as a control covariate in the insurance hypothesis analysis because SST and Chl-a were found to be similarly good proxies of food abundance (see Supplementary Material). Chl-a was omitted from the four cox models to avoid reducing sample size and statistical power, especially in broods of three, which are less common.

#### Facilitation hypothesis

First, we tested whether the presence of a last-hatched chick during development (through fledging at 70 d) positively affected first-hatched chicks’ body mass at fledging (70 d) in two-chick broods from cohorts 2003–2019; broods of three were too few for testing. We compared first-hatched fledglings that shared the nest with their junior sibling for at least 6 days (control group; *n* = 947) with first-hatched fledglings whose sibling was depredated (“experimental” group; *n* = 153). A linear mixed model was fitted with sibling presence (0 = no, 1 = yes), ulna length (in mm) at 70 days to control for body size, laying date, the values of SST, Chl-a, and rainfall during the windows that best-explained first-hatched chicks’ body mass as covariates (see Supplementary Material), and year as a random effect. Two interactions were also examined: sibling presence × SST and sibling presence × Chl-a.

We tested the effect of sibling presence during development on first-hatched fledglings’ probability of recruiting into the breeding population (being sighted with a partner, nest, and egg in any of the first 6 years) by fitting a generalized linear mixed model (GLMM) with binomial distribution (*n* = 1645 broods; model 1). We also evaluated the effect of sibling presence on the eventual mean laying dates of first-hatched fledglings (model 2) and the total of fledglings and recruits they produced (models 3 and 4) during their first 6 years of life. We analyzed using three GLMMs with a normal error distribution (model 2) and a Poisson distribution (models 3 and 4) the sub-sample of fledglings that recruited, from cohorts 1989-2013 for models 2 (*n* = 403 broods) and 3 (*n* = 424 broods), and from cohorts 1989-2007 for model 4 (*n* = 387 broods). Cohort 2007 was the most recent one for which we could count the number of recruits eventually produced by each individual. In addition to sibling presence, three covariates were included: laying date (to control for the hatching date of the focal individual), body condition at fledging (residuals from a linear regression of log body mass against log ulna length; for model 1), and sex (for models 2–4 as the sample included only sexed breeders). The cohort was included as a random effect to control for environmental conditions experienced during development. Because Blue-footed boobies are sexually dimorphic in size, the interaction between sibling presence and the sex of the focal bird was included in models 2–4 to test for a sexually asymmetric effect of sibling presence.

#### Quantifying the benefits of resource-tracking and insurance

The contribution of last-hatched offspring to parents’ reproductive success through resource-tracking (extra reproductive value) versus insurance was quantified separately in all broods of two (*n* = 5998) and three (*n* = 1310) established between 1989 and 2019. For this, we partitioned last-hatched chicks’ total reproductive value (RV_tot_) into extra reproductive value (RV_e_: its value when the entire brood survived) and insurance reproductive value (RV_i_: its value when it survived following the death of its older sibling), using Mock and Parker’s (1986) formula.

- RV_tot_ = RV_e_ + RV_i_
- RV_e_ = *q* * *P*_e_
- RV_i_ = (1—*q*)*P*_i_

Where *q* is the proportion of broods where an older sibling did not predecease a younger offspring, and *P*_e_ and *P*_i_ are, respectively, the fractions of *q* and (1—*q*) broods in which the last-hatched chick survived. To assess the impact of biotic and abiotic factors on the “extra” and “insurance” value of last-hatched offspring, we computed RV_e_ and RV_i_ for parents under various SST conditions during the incubation and brood care stages (i.e., the 110 days following the laying of the first egg) and for parents from different age-classes. In order to facilitate between-group comparisons, we grouped SST and age into categories that ensured large and equal sample sizes.

## RESULTS

### Resource-tracking hypothesis

**Figure 1:**
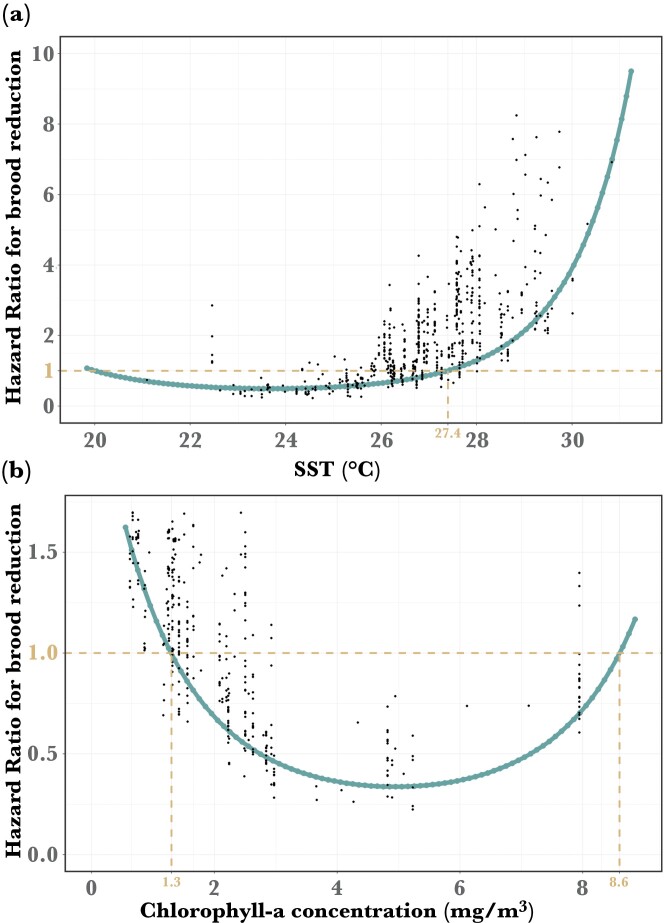
Effect of (a) minimum SST in a given week and (b) maximum Chl-a concentration in a given week on the hazard ratio (HR) for brood reduction; HR = 1 indicates no effect of SST/Chl-a on brood reduction probability (horizontal dashed line), HR > 1 indicates a positive effect, HR < 1 indicates a negative effect. Dots represent the predicted hazard ratio from the Cox model (*n* = 758 broods). Vertical dashed lines indicate (a) SST and (b) Chl-a values when HR = 1.

In broods of two, most brood reduction occurred during the first 4 weeks after hatching (Supplementary Figure S1), and in 76% of nests, it involved the death of the last-hatched chick. The probability of brood reduction was best explained by the weekly minimum SST and maximum Chl-a, 0.5 and 3 weeks prior to chick death, respectively (Supplementary Figure S2). As predicted, the likelihood of brood reduction increased as SST rose and Chl-a fell (SST: β ± SE = 0.57 ± 0.16, *n* = 1080, *P* < 0.001, Figure 1a; Chl: β ± SE = -1.15 ± 0.23, *P* < 0.001, Figure 1b). Both SST and Chl-a effects on brood reduction probability were quadratic (SST^2^: β ± SE = 0.36 ± 0.14, *P* = 0.01; Chl^2^: β ± SE = 0.42 ± 0.22, *P* = 0.05). Our model predicts sharp increase in brood reduction at SST over 27.4 °C (Figure 1a) and Chl-a below 1.3 mg/m^3^ or above 8.6 mg/m^3^ (Figure 1b). These adverse SST and Chl-a conditions were observed in 59% and 73% of weeks, respectively, throughout the reproductive seasons of 2003 through 2019.

In females, brood reduction likelihood decreased with age through 11 years and increased with age thereafter (age: β ± SE = -0.47 ± 0.17, *P* < 0.01, age^2^: β ± SE = 0.51 ± 0.16, *P* < 0.01, Supplementary Figure S3) whereas male age had no effect (*P* = 0.19).

### Insurance hypothesis

In broods of three, as predicted, the survival probability of the last-hatched chick was higher when either of its older siblings died, while the survival of the first-hatched chick was unaffected by the death of either younger sibling (Table 2). In these broods, more than half of first- and last-hatched chick deaths occurred during the first 20 and 6 days of life, respectively (Supplementary Figure S5c, d).

However, contrary to our prediction, in broods of two, independent of a chick’s rank, the death of its sibling increased its risk of death by 71% (Table 1). After the death of their only sibling, 39% of first-hatched chicks and 52% of last-hatched chicks died within 6 days, hinting at nest desertion by parents. More than half of first- and second-hatched chick deaths in broods of two occurred during the first 15 days of life (Supplementary Figure S5a, b).

**Table 1:** Factors affecting survival probability of first- and last-hatched chicks in broods of two

Terms	First-hatched chick (*n* = 1948)				Last-hatched chick (*n* = 1766)			
	β	SE	*Z*	*P*	β	SE	Z	*P*
Sibling death	**1.53**	**0.13**	**11.78**	**<0.001**	**0.51**	**0.13**	**3.93**	**<0.001**
Laying date	0.20	0.15	1.37	0.170	**0.83**	**0.12**	**6.72**	**<0.001**
Hatching interval	0.16	0.08	1.92	0.055	**0.26**	**0.05**	**4.87**	**<0.001**
SST	**0.81**	**0.14**	**5.85**	**<0.001**	**0.65**	**0.11**	**5.93**	**<0.001**
SST²	**0.24**	**0.09**	**2.61**	**0.009**	**0.33**	**0.09**	**3.78**	**<0.001**
Rain	**1.02**	**0.23**	**4.38**	**<0.001**	**0.93**	**0.20**	**4.70**	**<0.001**
Rain²	**−0.14**	**0.06**	**−2.51**	**0.012**	**−0.16**	**0.05**	**−3.09**	**0.002**
		First-hatched chick		Last-hatched chick				
Random effects	σ	σ²	σ	σ²				
Year	0.58	0.34	0.66	0.43				
Nest ID	0.02	0.00	0.02	0.00				

The “-” symbol means that the interaction term was removed from the final model as it did not improve model fit (by comparing AICc). Statistically significant terms are in bold.

In both brood sizes, long hatching intervals between first- and last-hatched chicks were detrimental only to the latter, which in our samples were significantly less likely to fledge (Tables 1 and 2).

**Table 2:** Factors affecting survival probability of first- and last-hatched chicks in broods of three

Terms	First-hatched chick (*n* = 818)				Last-hatched chick (*n* = 701)			
	β	SE	*Z*	*P*	β	SE	*Z*	*P*
Sibling death	−0.56	0.62	−0.91	0.363	−**0.58**	**0.24**	−**2.43**	**0.015**
Laying date	**0.89**	**0.40**	**2.25**	**0.025**	**0.97**	**0.12**	**8.43**	**<0.001**
Hatching interval	0.03	0.39	0.07	0.940	**0.54**	**0.11**	**4.98**	**<0.001**
SST	0.02	0.44	0.05	0.964	−0.03	0.15	−0.18	0.854
SST²	0.32	0.26	1.20	0.229	−0.02	0.15	−0.17	0.868
Rain	−0.58	1.28	−0.45	0.651	−0.03	0.23	−0.12	0.902
Rain²	0.03	0.22	0.14	0.891	0.00	0.01	0.08	0.940
	First-hatched chick		Last-hatched chick					
Random effects	σ	σ²	σ	σ²				
Year	0.68	0.46	0.81	0.66				
Nest ID	0.02	0.00	0.10	0.01				

Statistically significant terms are in bold.

### Facilitation hypothesis

**Figure 2:**
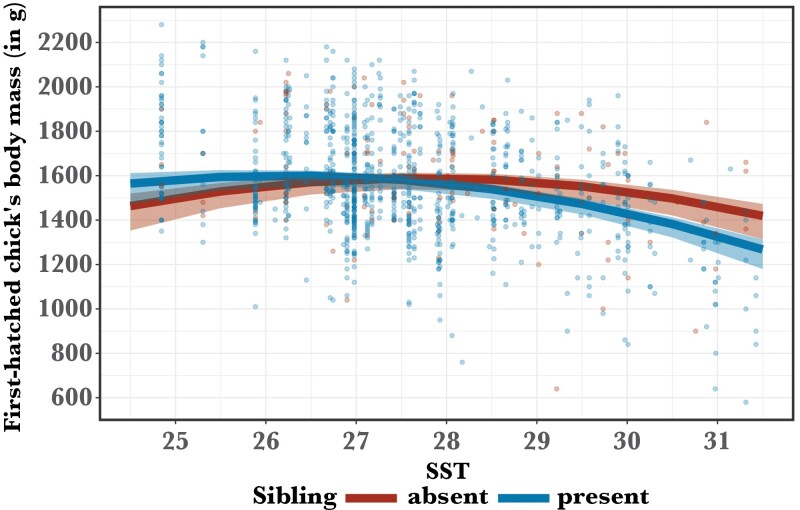
Interaction effect of SST and sibling presence on first-hatched chick’s body mass controlled for body size at 70 days (*n* = 1100 broods). Shaded areas depict 95% confidence intervals.

Contrary to predictions, sibling presence during development did not improve the body condition of first-hatched fledglings (*n* = 1100, *P* = 0.52, Supplementary Table S2), and condition of first-hatched offspring that cohabited with their sibling for at least 6 days was more adversely affected by high SST than that of their single counterparts in depredated broods (SST: β ± SE = 5.96 ± 35.61; sibling presence × SST: β ± SE = −95.12 ± 31.8, *n* = 1100, *P* < 0.01; Figure 2). The results remained consistent when sample size was increased to 1589 first-hatched fledglings by using only SST as a proxy for food abundance. Likewise, sibling presence did not improve recruitment probability or reproductive parameters (average laying date, numbers of fledglings and recruits produced) during the first 6 years after fledging, although the statistical power of the last three tests (models 2, 3, and 4) was relatively low (all GLMMs: *n* ≥ 316, *P* ≥ 0.08, Supplementary Table S3).

Similarly, in the control group, the duration of cohabitation had no effect on first-hatched chicks’ body condition, recruitment, or reproductive parameters (see Supplementary Tables S4 and S5).

### Contributions of resource-tracking and insurance to reproductive value

In broods of two and three, approximately 90% of the contribution of last-hatched chicks to parental reproductive success (RV_tot_) was via extra reproductive value (RV_e_) and only ≈ 10% via insurance reproductive value (RV_i_) (Table 3).

**Table 3:** Extra (RV_e_) and insurance (RV_i_) reproductive value of last-hatched chicks in broods of two and three with their associated 95% bootstrap confidence intervals (calculated with 1e4 iterations)

	Brood size			
	2 (*n* = 5998)		3 (*n* = 1310)	
Reproductive value	Value	CI	Value	CI
RVe	0.605	0.592 | 0.618	0.434	0.407 | 0.461
RVi	0.057	0.051 | 0.063	0.051	0.04 | 0.063

**Figure 3:**
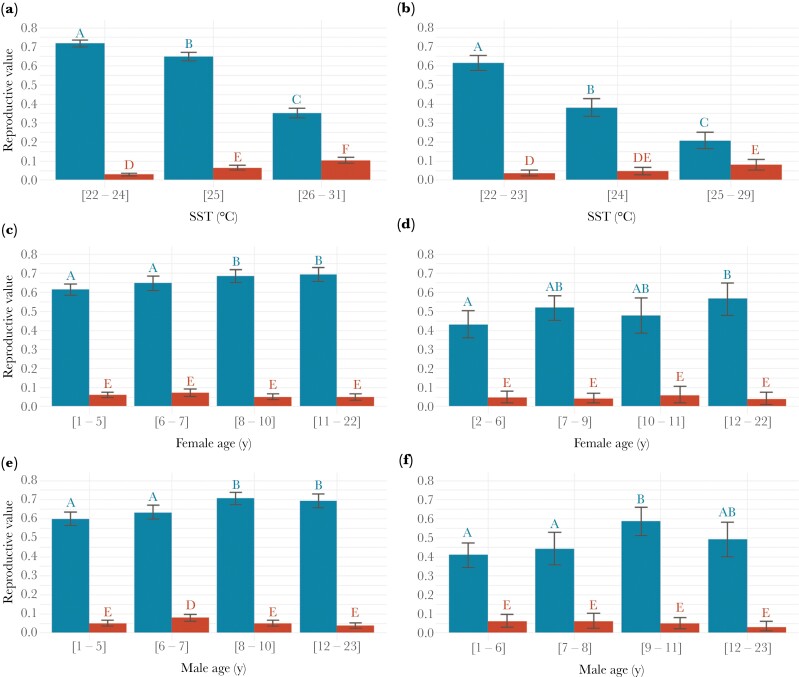
Extra (RV_e_, *blue*) and insurance (RV_i_, *red*) reproductive value of last-hatched chicks in broods of two (*left*) and three (*right*) in relation to SST and parents’ ages. Error bars represent the 95% bootstrap confidence intervals (calculated with 1e4 iterations). SST is the average sea surface temperature experienced by the parents over the 110 days following laying of the first egg (i.e., incubation and brood care stages). Different letters indicate a significant difference (*P* < 0.05, 95% CI of the difference between groups does not encompass zero). Sample sizes were: *n* = 5935 broods for a, *n* = 1302 broods for b, *n* = 3312 broods for c, *n* = 689 broods for d, *n* = 3298 broods for e, *n* = 703 broods for f.

In both brood sizes, as SST rose, RV_e_ of last-hatched chicks decreased while their RV_i_ increased (Figure 3a,b). Independent of brood size, RV_e_ tended to increase with parents’ ages (Figure 3c,d,e,f). In male parents of broods of three, RV_e_ appeared to peak in middle-aged individuals between 8 and 11 years old (Figure 3e,f). Conversely, RV_i_ did not follow a consistent trend among parents’ sex or brood size, remaining mostly constant across parents’ ages (Figure 3c,d,e,f).

## DISCUSSION

### Resource-tracking hypothesis

Monitoring of two environmental variables and hundreds of booby nests over a period of 30 years including several El Niño oscillations, confirmed that overproduction of nestlings functions as a hedge against resource stochasticity. Overproduction allows Blue-footed boobies to opportunistically fledge extra chicks when food is abundant. However, when food is scarce, as indexed by high SST (>27 °C; Figure 1a) and low Chl-a (<1.3 mg/m^3^; Figure 1b), boobies rapidly reduce their broods.

In the Gulf of California, SST associates more strongly with fish abundance in the following two weeks rather than the current abundance (Lanz et al. 2009). Warm SST is generally associated with ocean stratification, which limits the upwelling of deep, cold, and nutrient-rich water, hampering phyto- and zooplankton blooms (Dunstan et al. 2018). Hence at Isla Isabel, ≈350 km south of the Gulf, rapid brood reduction (within 1 week) following very high SST could be an anticipatory response by parents, moderating feeding effort to accelerate siblicide, as suggested for other siblicidal seabirds (Leclaire et al. 2010). An anticipatory response could be adaptive in long-lived brood-reducing species, with rapid death of last-hatched chicks diminishing current parental effort in favor of future reproduction (Vedder et al. 2017). Alternatively, booby brood reduction could be an immediate response to poor food conditions accompanying high temperature and, indeed, in the Coral Sea, Australia, warm SST is thought to directly influence the spatial distribution of fish by making them less accessible to seabirds (Peck et al. 2004; Erwin and Congdon 2007). Independent of the underlying mechanism, brood reduction in warm water conditions reveals seabirds’ vulnerability to short-term climate variation, and implies that the imminent increase in frequency of marine heat waves will precipitate more frequent brood reduction (Coumou and Rahmstorf 2012).

Low Chl-a is correlated with low current pelagic fish abundance as it reflects a poor feeding environment for planktivorous fish (Lanz et al. 2009). Paradoxically, the probability of brood reduction also appeared to increase at the highest Chl-a concentrations (>8.6mg/m^3^; Figure 1b), possibly in response to the hypoxic (low-oxygen) conditions, which are correlated with surface Chl-a concentrations and deplete pelagic fish populations (Diaz and Rosenberg 1995). In the Gulf of California, severe hypoxia has been documented, in association with extreme phytoplankton blooms caused by agricultural runoffs (reviewed in Lluch-Cota et al. 2007). Hence, Chl-a, unlike SST, is an ambiguous index of pelagic fish abundance.

### Insurance hypothesis

Evidence for the insurance benefit, increased survival of last-hatched chicks after the death of elder siblings, was found only in broods of three (Table 2), where last-hatched chicks replaced siblings that died, thus preventing brood size from falling below parents’ provisioning capacity (Mock and Forbes 1995). Moreover, since last- and first-hatched fledglings of two-chick broods have almost equivalent fitness in this species (Drummond et al. 2003; Drummond and Rodríguez 2013), the quality of the fledged brood should be maintained after elder chicks are substituted by last-hatched chicks. Finally, as also predicted by the insurance hypothesis, the survival of first-hatched chicks in three-chick broods was unaffected by the death of a younger sibling, reflecting efficient brood reduction through competitive asymmetry (Stoleson and Beissinger 1997).

In contrast, the death of a chick in broods of two reduced the survival of its sibling independently of the chick’s rank (Table 1). There are two likely causes for this discrepancy between two- and three-chick broods. First, poor-quality parents (e.g., poor at foraging, reading environmental cues, or brooding) may sometimes be unable to fledge even one of their two chicks, a situation likely to be rare in broods of three because parents of larger broods tend to be high-quality individuals (Townsend and Anderson 2007). Second, the early death of a chick could cue a parental strategy of adaptive abandonment (Mock and Parker 1986; Ackerman and Eadie 2003; Kupán et al. 2021) that maximizes parents’ fitness by improving their future reproductive success (McKnight et al. 2018). Parents should abandon whenever the cost in relation to the current brood is exceeded by the increment in future reproductive success (Mock and Parker 1986; Székely et al. 1996), particularly in long-lived birds like Blue-footed boobies whose lifetime fitness can be seriously impacted by a reduction in survival (Stearns 1992).

Interestingly, a strategy of parental abandonment after loss of one of two chicks could constrain selection for unnecessary siblicidal brood reduction, with last-hatched chick presence stimulating parents to maintain provisioning of the brood (Mock and Parker 1986). Similarly, in broods of three, desertion could also be expected following the death of both senior chicks. The latter is so uncommon that we could not test the idea, but we note that in 17 nests where both seniors died, 58% of surviving third-hatched chicks died within 6 days.

Note that this study tested the insurance function of last-hatched offspring only at the chick stage. We could not assess the insurance value of extra eggs as a hedge against egg predation or hatching failure because eggs were not marked, and laying order was unknown prior to hatching. As egg predation by gulls is common, zygote overproduction for insurance against egg loss is highly likely.

### Facilitation hypothesis

Our brood size “experiment” did not support the main prediction of the facilitation hypothesis. First-hatched chicks that shared the nest with their sibling were not in better condition than those whose siblings were depredated, and even had a slightly worse body condition than them during warm SST (Figure 2). As parents of siblicidal Pelecaniformes tend to reduce per capita provisioning following brood reduction (Ploger 1997), first-hatched booby chicks likely received less food after a sibling was depredated. However, they grew just as well, possibly because they ceased engaging in aggressive sibling rivalry, which is expected to be costly (Ostreiher and Heifetz 2016).

The pseudo-experimental approach also failed to detect any long-term benefits of cohabiting with a junior sibling (Edwards 1989; Bebbington et al. 2017), regarding the probability of recruitment, earliness of laying, or numbers of fledglings and recruits produced during the first 6 years of life. It has been speculated that selective mortality during the nestling period could obscure evidence of sibling facilitation by allowing only high-quality individuals of reduced or unreduced broods to fledge and recruit into the breeding population (Cam and Monnat 2000). In broods of two booby chicks, nearly one-third of first-hatched offspring fail to fledge, so selective filtering is a possibility (Drummond et al. 2011). However, filtering would bias our test in favor of finding spurious facilitation, and this did not occur.

### Extra and insurance value of last-hatched offspring

Resource tracking was clearly the primary function of parental overproduction, as RV_e_ accounted for 90% of the total reproductive value of last-hatched chicks (Table 3). Last-hatched chicks were 36% more valuable to parental fitness in broods of two than in broods of three (RV_e_; Table 3), reflecting the lower likelihood of brood reduction in two-chick broods, which require less provision and compete less intensely (Valderrábano-Ibarra et al. 2007). On the other hand, the RV_i_ of last-hatched chicks was similarly low in both brood sizes (Table 3), contrary to Forbes’s (1990) prediction of higher RV_i_ in larger avian broods. However, RV_i_ comprises last-hatched chicks that survived because their older sibling died plus last-hatched chicks that would have survived even if their older sibling had not died (Lamey et al. 1996). Hence, our estimates of RV_i_ are inflated by the inclusion of the latter, particularly in broods of two where last-hatched chicks are less dependent on first-hatched chick outcome, obscuring the expected result.

The extra and insurance value of last-hatched offspring varied differently with biotic and abiotic factors (Figure 3). As SST rose during incubation and brood care, the RV_e_ of last-hatched chicks decreased, possibly reflecting parents’ struggle to predict food availability during chick-rearing. In contrast, RV_e_ in American kestrel broods remained stable across years despite substantial inter-annual variation of prey abundance. Kestrels may be better able to forecast food abundance and avoid brood reduction (Wiebe 1996), likely as they prey on small mammals whose populations change slowly over the season (Forbes et al. 2001). Alternatively, interannual variation in RV_e_ of boobies could be greater because poor-quality individuals or first-time breeders, more prone to chick failure (Riotte-Lambert and Weimerskirch 2013) might be overrepresented among breeders during poor conditions as intrasexual competition might be lower (Cubaynes et al. 2011). On the other hand, an increase in the insurance value of last-hatched chicks under high SST could derive from poor-quality boobies breeding during high SST, or from fewer adults initiating reproduction during poor environmental conditions (Ancona et al. 2011), making nests more likely to suffer depredation.

Finally, RV_e_ of the last-hatched chick increased with the age of both parents, possibly due to benefits of experience (Daunt et al. 2007), selective survival of higher quality adults, and/or decreasing restraint on reproductive effort as prospects for future reproduction decrease (Stearns 1992). Interestingly, the probability of successfully rearing the whole brood of three (RV_e_) peaked in middle-aged fathers (Figure 3e,f). This peak could be linked to senescence, widely documented in male boobies beyond 10 years, due to a decline in their sexual attractiveness limiting access to high-quality mates (Torres and Velando 2003) and/or eliciting reduction in female partners’ parental investment (Velando et al. 2006; Torres and Velando 2007). Furthermore, senescent males could sire progeny with genetic disorders due to DNA damage in germinal cells (Sartorius and Nieschlag 2010; Velando et al. 2011).

Our data supported two of three proposed functions of parental overproduction. Last-hatched young mainly contributes to parents’ fitness by allowing them to fledge extra chicks under favorable food conditions (resource-tracking) and, to a lesser degree, by replacing failed chicks in broods of three (insurance). Our results also underscore the importance of considering short-term environmental variability in addition to longer-term fluctuations like ENSO, as they impinge strongly on boobies’ reproductive success. As ocean productivity declines with global warming (Behrenfeld et al. 2006) and heatwave frequency increases, we should expect parental overproduction of chicks to lose some of its functionality through progressive erosion of its resource-tracking benefit. Reduction of overproduction’s benefits while its costs to parents’ condition and future reproduction (Velando and Alonso-Alvarez 2003) persist or increase could have demographic consequences. Whether this would select for smaller clutch sizes in the long or short term is unclear because extra eggs might also buffer a booby’s nesting attempt against the loss of single eggs, and clutch size plasticity in this species is yet to be fully understood.

## Supplementary Material

arae007_suppl_Supplementary_Data

arae007_suppl_Supplementary_Figure

## Data Availability

Analyses reported in this article can be reproduced using the data provided by Bizberg-Barraza et al. (2024).
