# Supplementary material for: Parental overproduction allows siblicidal bird to adjust brood size to climate-driven prey variation
Source: Behav Ecol. 2024 Feb 1;35(2):arae007. doi: 10.1093/beheco/arae007 (PMC10878367; doi:10.1093/beheco/arae007)
Supplement: arae007_suppl_Supplementary_Data [file arae007_suppl_supplementary_data.docx]

**Supplementary Material: Parental overproduction allows siblicidal bird to adjust brood size to climate-driven prey variation**

# Resource tracking hypothesis

## Adjustment of brood size to environmental conditions


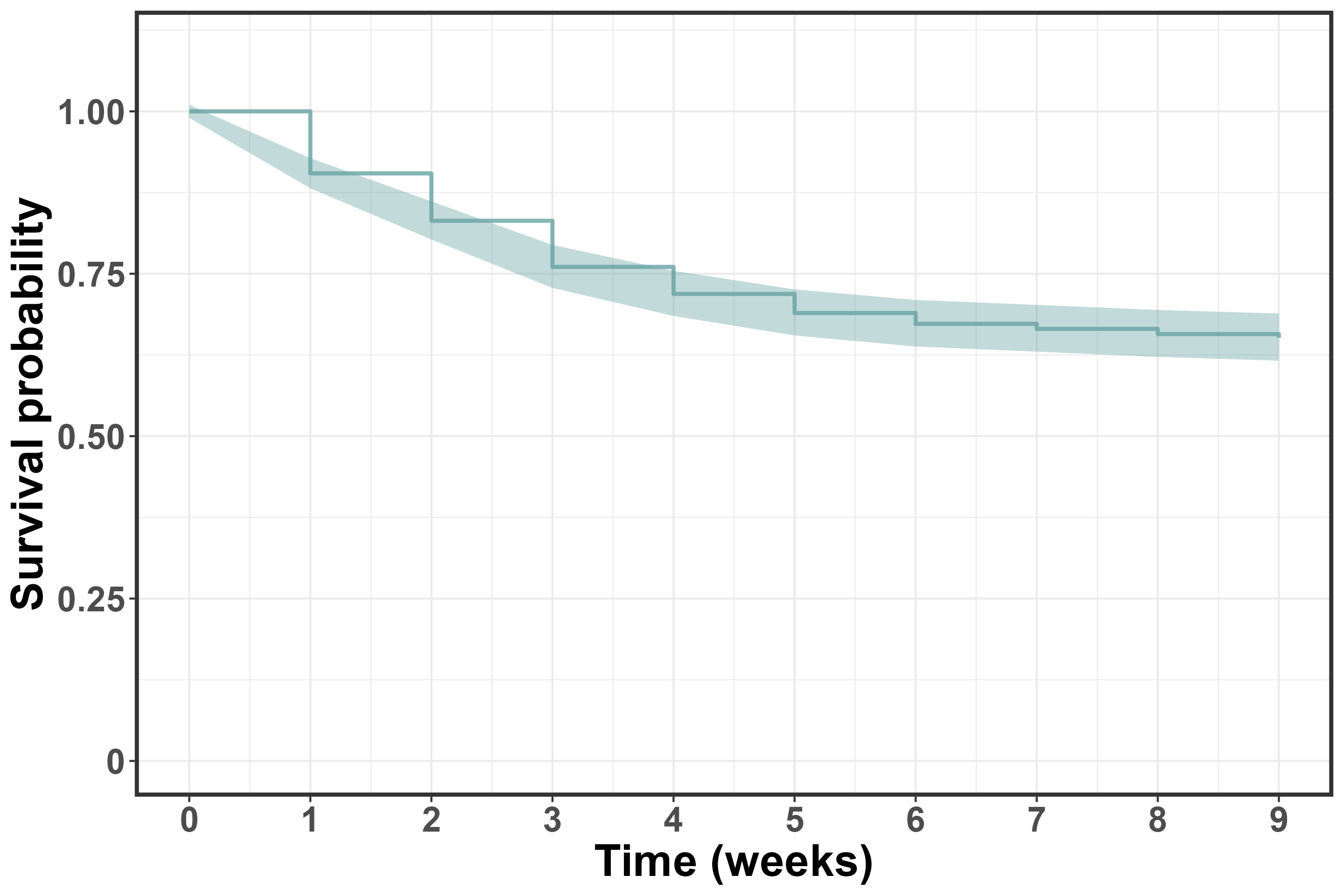

**Figure S1**: Survival curves of both chicks in the nest. The y-axis indicates the survival probability of both chicks in the nests (N = 758 broods). The x-axis is the time in weeks since the hatching of the entire brood.


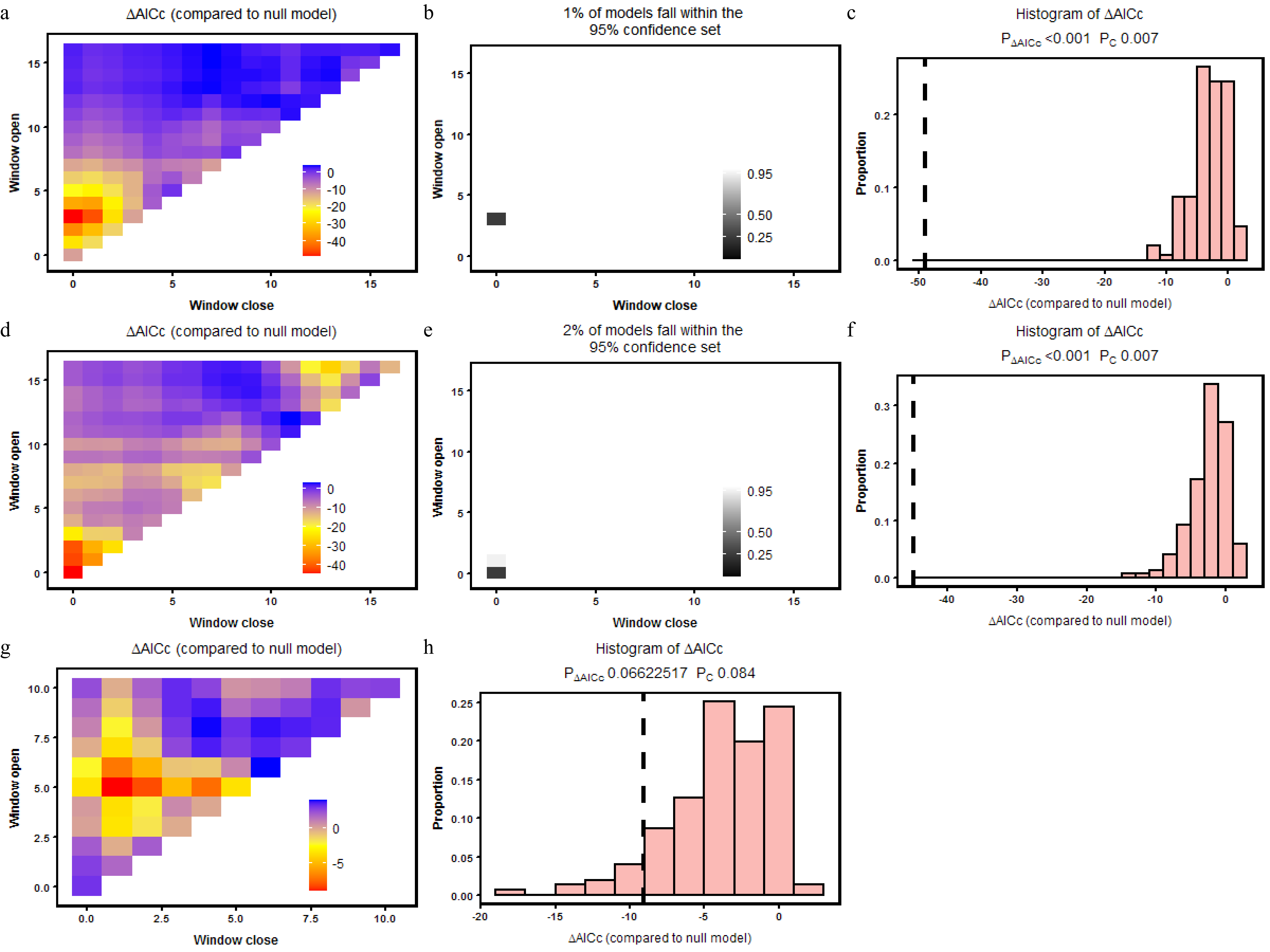

**Figure S2**: Output of sliding window analysis for the effects of environmental conditions on the likelihood of brood reduction. Chl-a concentration and SST, but not rainfall, influenced brood reduction (a-h). Model support (∆AICc) for all tested combinations of opening (y-axis) and closing (x-axis) windows, in weeks before brood outcome (brood was reduced or both chicks fledged), for Chl-a (a), SST (d), and rainfall (g). The regions in red correspond to the windows that are the best-supported models, lowest ΔAICc values compared to the null model (model without environmental variables). Akaike model weights for relevant variables, Chl-a (b) and SST (e), showing in grey, the area where we can be 95% confident that the best model falls. Results of the randomization analyses for Chl-a(c), SST(f), and rainfall(h). Histogram show all ∆AICc values from the models fitted on the randomized data. The dashed line shows the ∆AICc of the best model fitted on the observed data. The probability that the signal is caused by chance increases when the ∆AICc of the best model (dashed line) overlaps with the randomized simulated results (histogram).


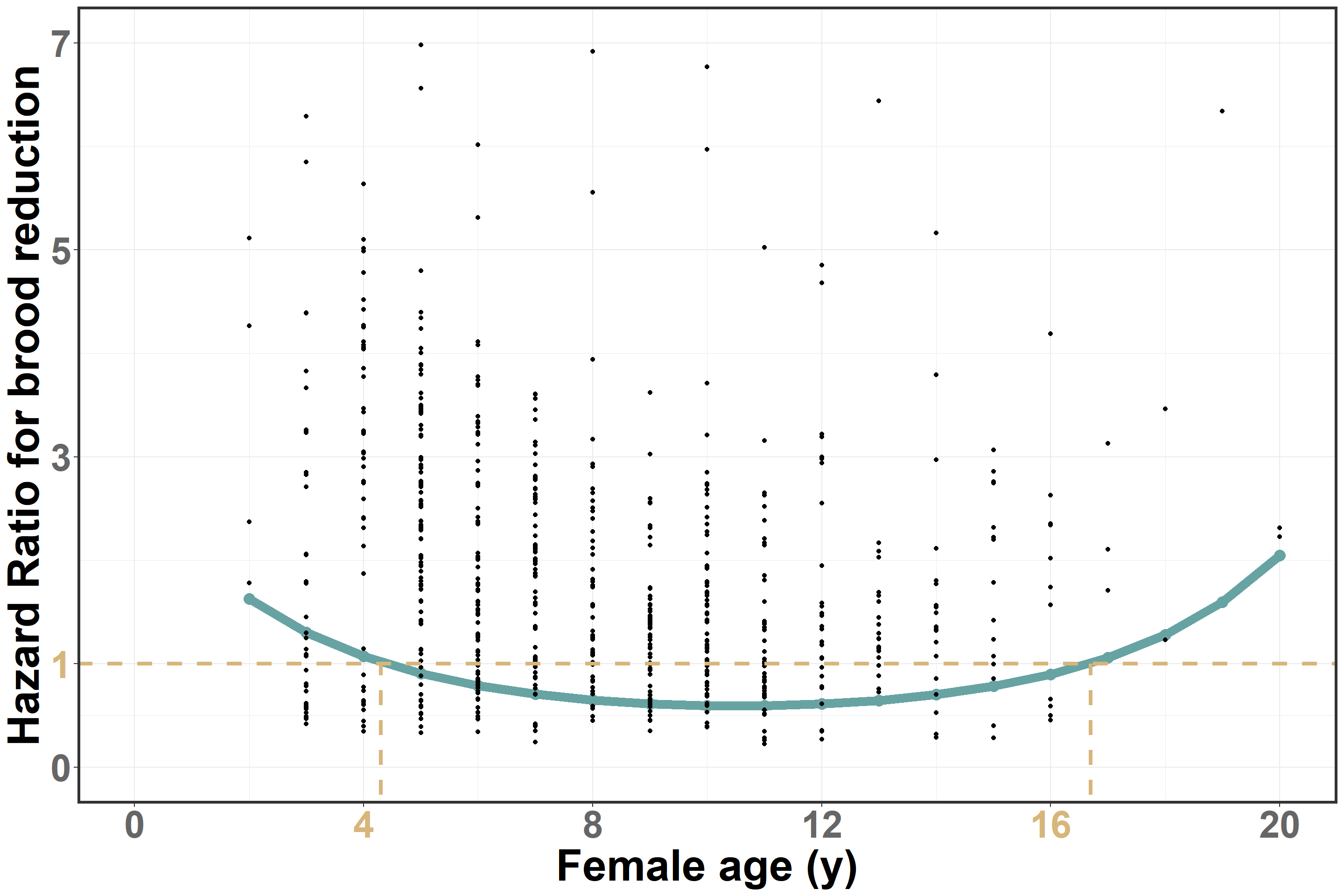

**Figure S3**: Effect of mother’s age on brood reduction’s hazard ratio (HR); HR = 1 indicates a null effect (horizontal dashed line), HR > 1 indicates a positive effect, HR < 1 indicates a negative effect. Dots represent the predicted hazard ratio from the cox model (N = 758 broods). Vertical dashed lines indicate female ages when HR = 1.

## Proxies of food availability and chick growth

To test whether SST and Chl-a are adequate proxies of food availability, we tested (separately in first- and second-hatched chicks) whether those variables had, respectively, a negative and a positive relationship with chicks’ body mass at 70 days old. All broods from cohorts 1989 to 2019 were used (first-hatched chicks: n = 5355; second-hatched chicks: n = 2921). The “climwin” baseline model, used to identify salient time windows, was a linear mixed model to which laying date, ulna length (mm), and cohabitation time (i.e., sum of shared days with their siblings) were added as covariates to control for intra-seasonal variation in food availability, chicks’ body size and sibling relationship effects. Year was included as a random effect. Based on preliminary analysis, the sliding approach was conducted for SST, Chl-a, and rainfall over a time window of 17 weeks prior to chick measurement.

      As expected from adequate proxies of food availability, the fledgling’s body mass was negatively affected by SST (during the 1-13 weeks prior to first-hatched chicks’ measurements and during the 14 weeks prior to second-hatched chicks’ measurements) and positively affected by Chl-a (3.5 weeks prior to first-hatched chicks’ measurements and 10 to 1 week for second-hatched chicks) (Table S1a, b; Figure S4a, b). Both environmental variables had a quadratic relationship with the fledgling’s body mass (Table S1a, b).


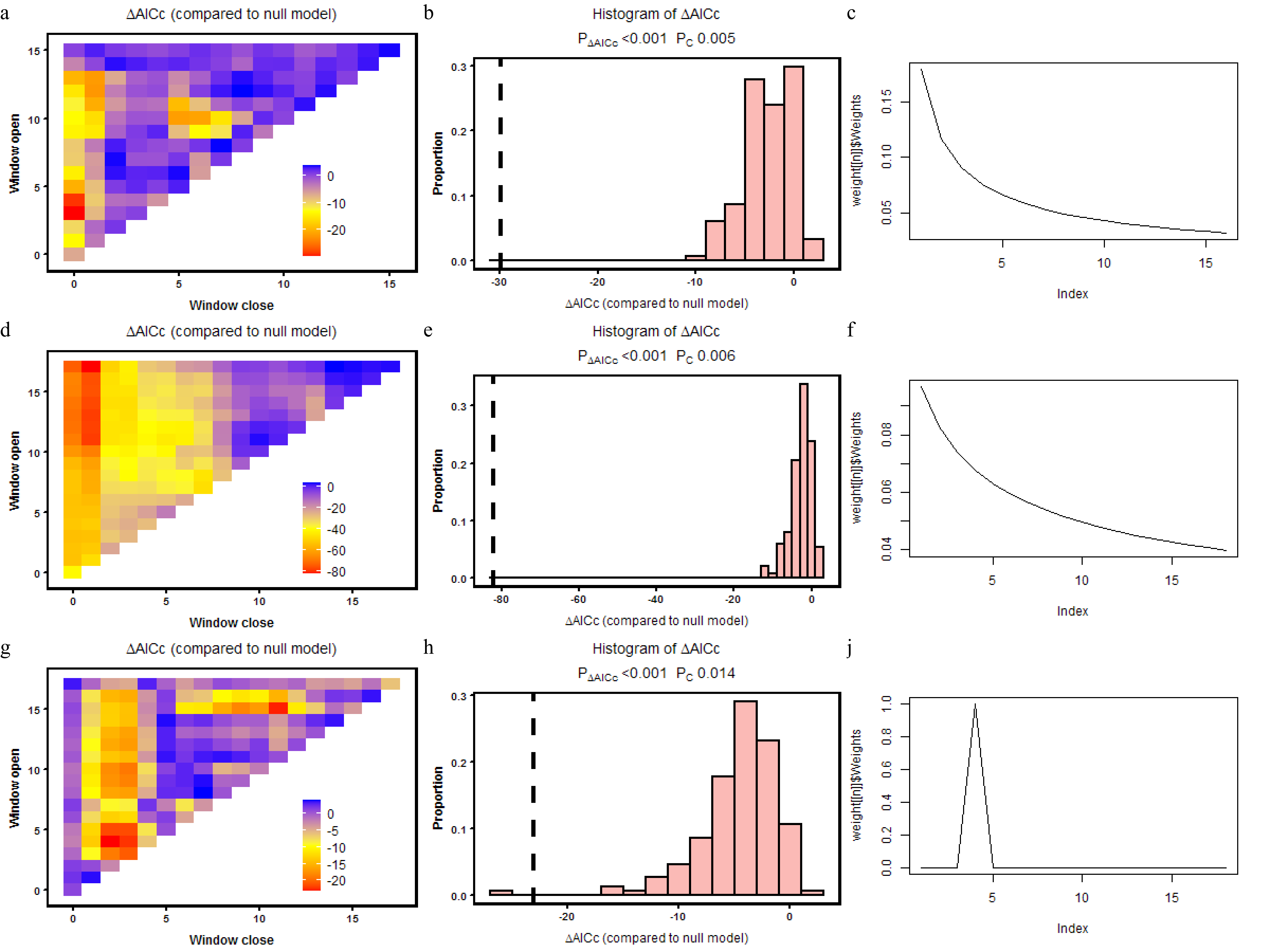

**Figure S4 a**: Output of sliding window analysis for the effects of environmental conditions on A-chick’s body mass at 70 days old. Chl-a concentration, SST, and rainfall influenced A-chick’s body mass (a-j). Model support (∆AICc) for all tested combinations of opening (y-axis) and closing (x-axis) windows, in weeks prior to chick measurement, for Chl-a (a), SST (d), and rainfall (g). The regions in red correspond to the windows that are the best-supported models, lowest ΔAICc values compared to the null model (model without environmental variables). Results of the randomization analyses for Chl-a(b), SST(e), and rainfall(h). Histogram show all ∆AICc values from the models fitted on the randomized data. The dashed line shows the ∆AICc of the best model fitted on the observed data. The probability that the signal is caused by chance increases when the ∆AICc of the best model (dashed line) overlaps with the randomized simulated results (histogram). Best weighted time window showing the influence of Chl-a(c), SST(f), and rainfall(j) in chick’s body mass across the tested time window (index). As the weight increase (y-axis), the week has a more critical effect on the response variable.


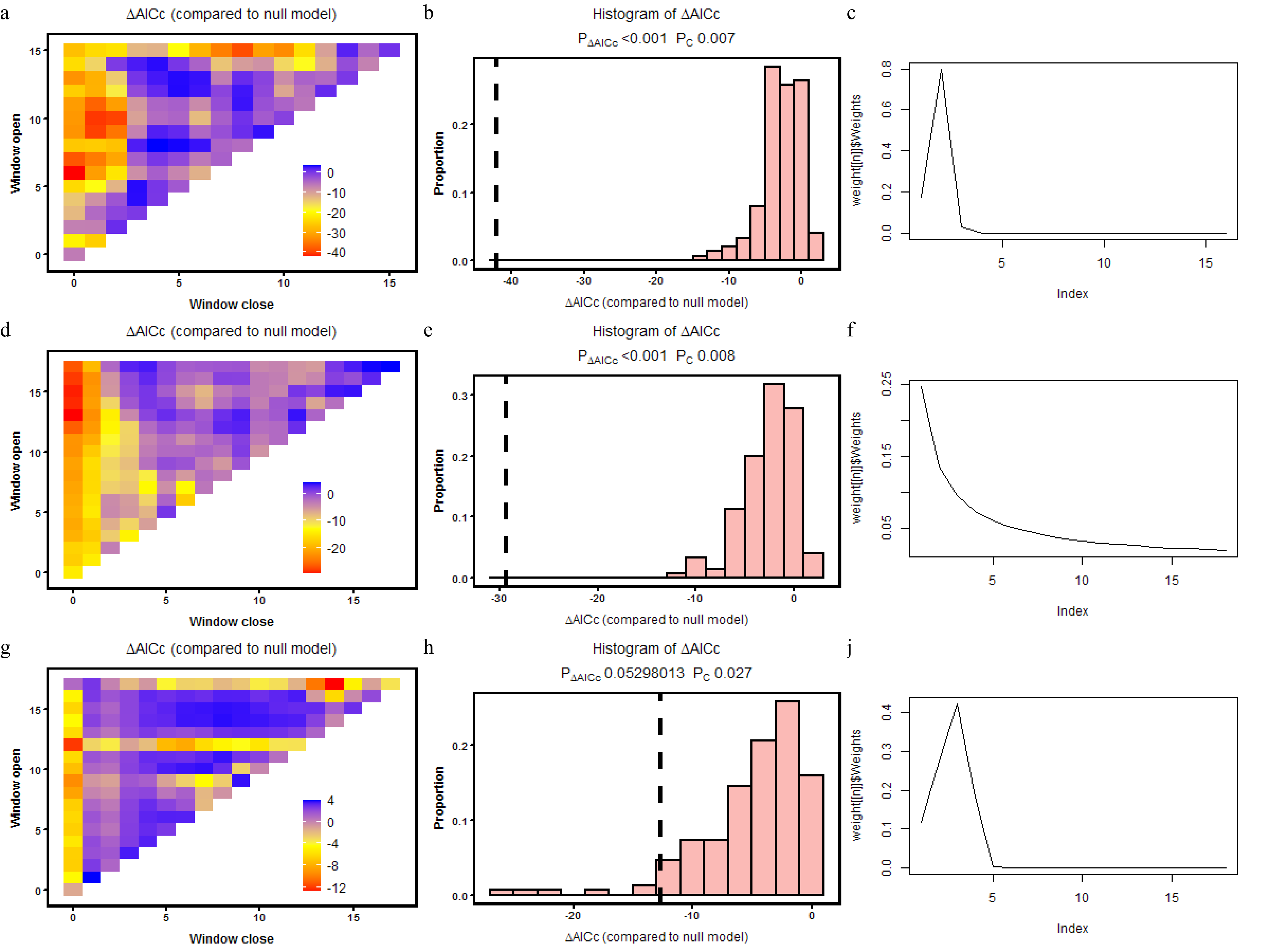

**Figure S4 b**: Output of sliding window analysis for the effects of environmental conditions on B-chick’s body mass at 70 days old. Chl-a concentration, SST, but not rainfall, influenced B-chick’s body mass (a-j). Model support (∆AICc) for all tested combinations of opening (y-axis) and closing (x-axis) windows, in weeks prior to chick measurement, for Chl-a (a), SST (d), and rainfall (g). The regions in red correspond to the windows that are the best-supported models, lowest ΔAICc values compared to the null model (model without environmental variables). Results of the randomization analyses for Chl-a(b), SST(e), and rainfall(h). Histogram show all ∆AICc values from the models fitted on the randomized data. The dashed line shows the ∆AICc of the best model fitted on the observed data. The probability that the signal is caused by chance increases when the ∆AICc of the best model (dashed line) overlaps with the randomized simulated results (histogram). Best weighted time window showing the influence of Chl-a(c), SST(f), and rainfall(j) in chick’s body mass across the tested time window (index). As the weight increase (y-axis), the week has a more critical effect on the response variable.


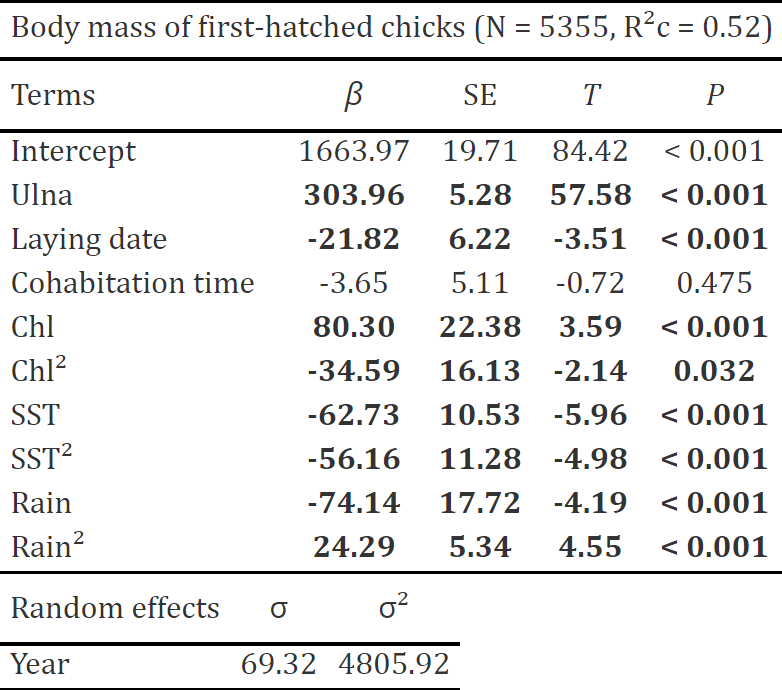

**Table S1 a**: Effects of Chl-a concentration and SST on the body mass of first-hatched chicks. Statistically significant terms are in bold.


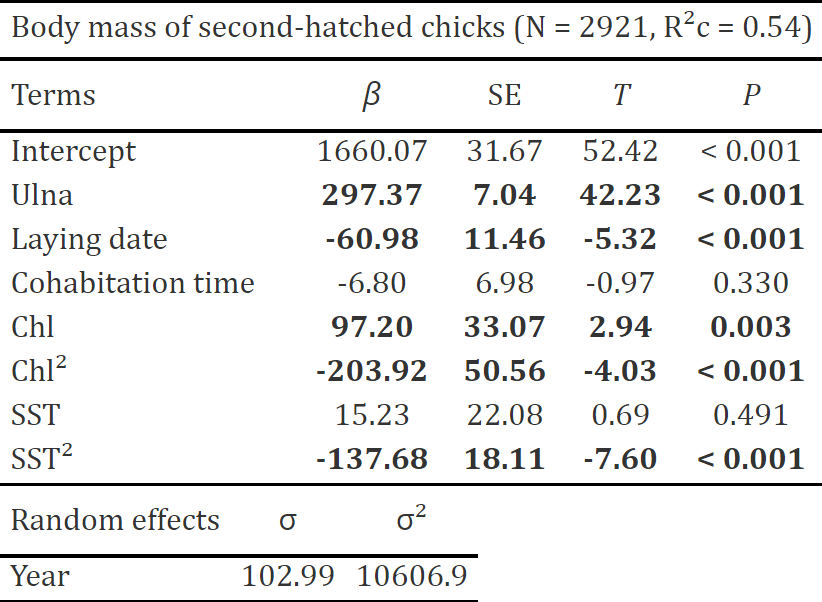

**Table S1 b**: Effects of Chl-a concentration and SST on the body mass of second-hatched chicks. Statistically significant terms are in bold.

# Insurance hypothesis


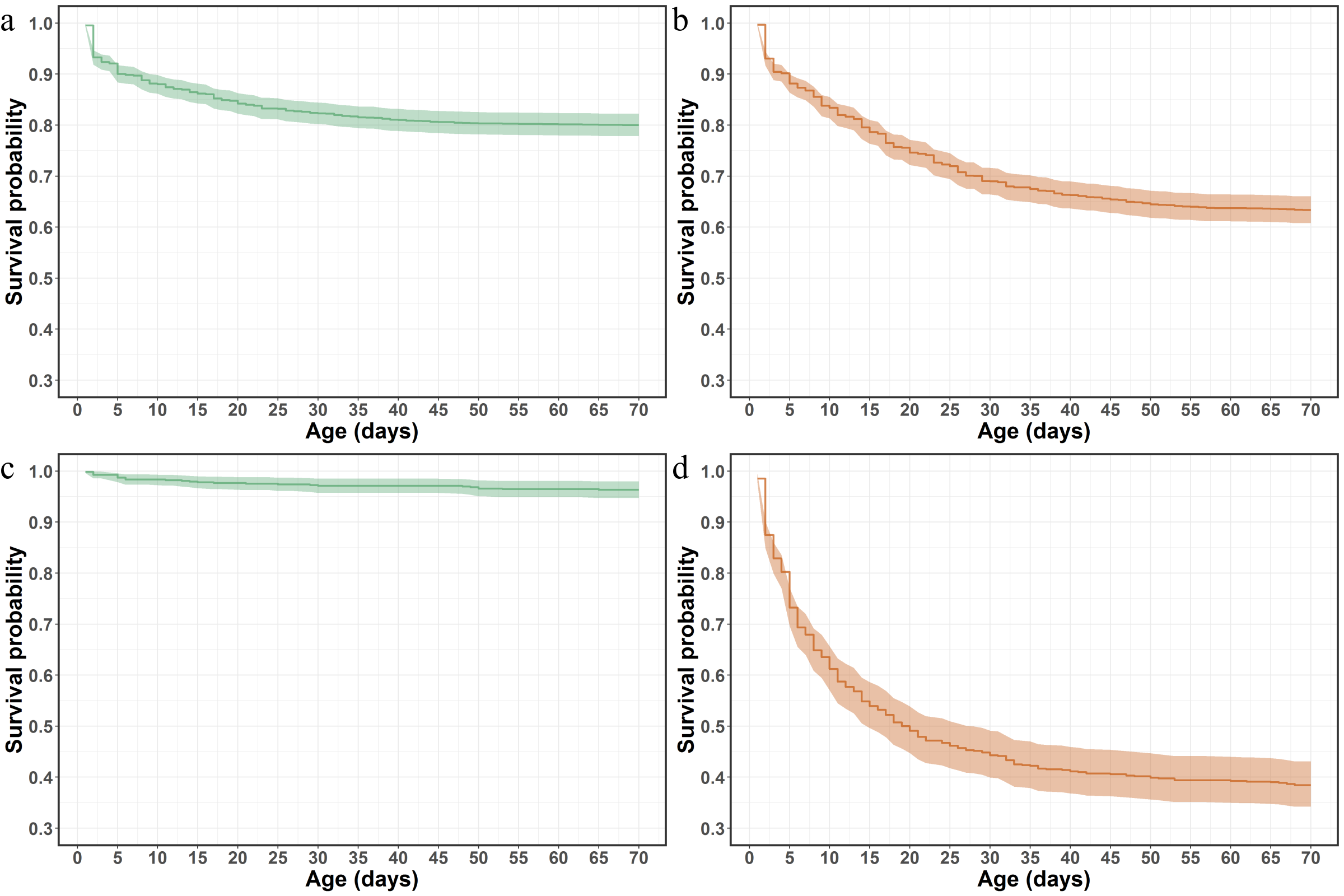

**Figure S5**: Survival curves of first-hatched chicks from broods of two (a; n = 1948) and three (c; n = 818), and survival curves of last-hatched chicks from broods of two (b; n = 1766) and three (d; n = 701). The y-axis indicates chicks’ cumulative survival probability at each age.

# Facilitation hypothesis


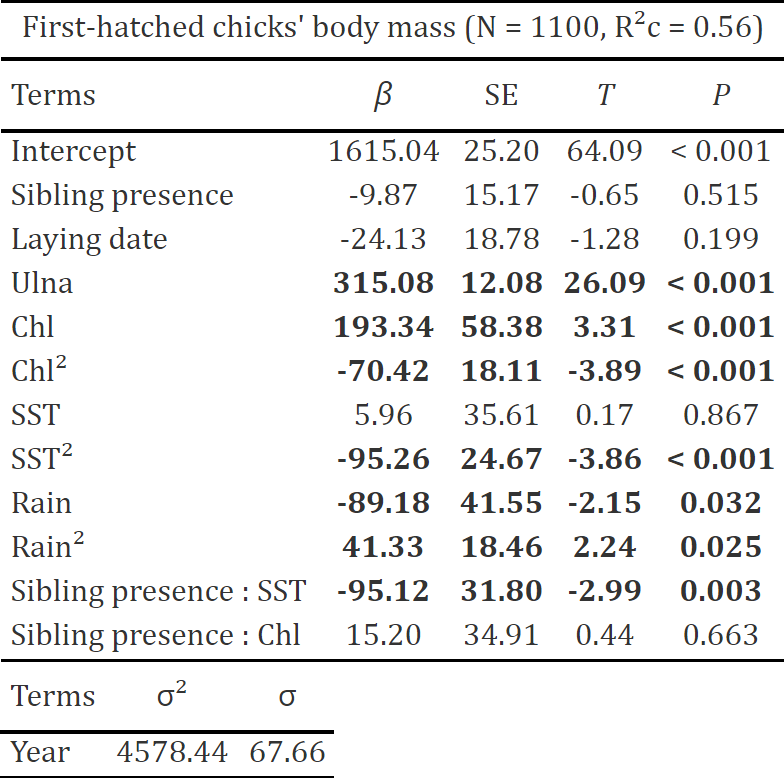

**Table S2**: Effect of sibling presence on core chick’s body mass at 70 days in broods of 2 chicks. Interaction effect of Chl-a and sibling presence on core chick’s body mass was dropped because it did not improve model fit. Statistically significant terms are in bold.


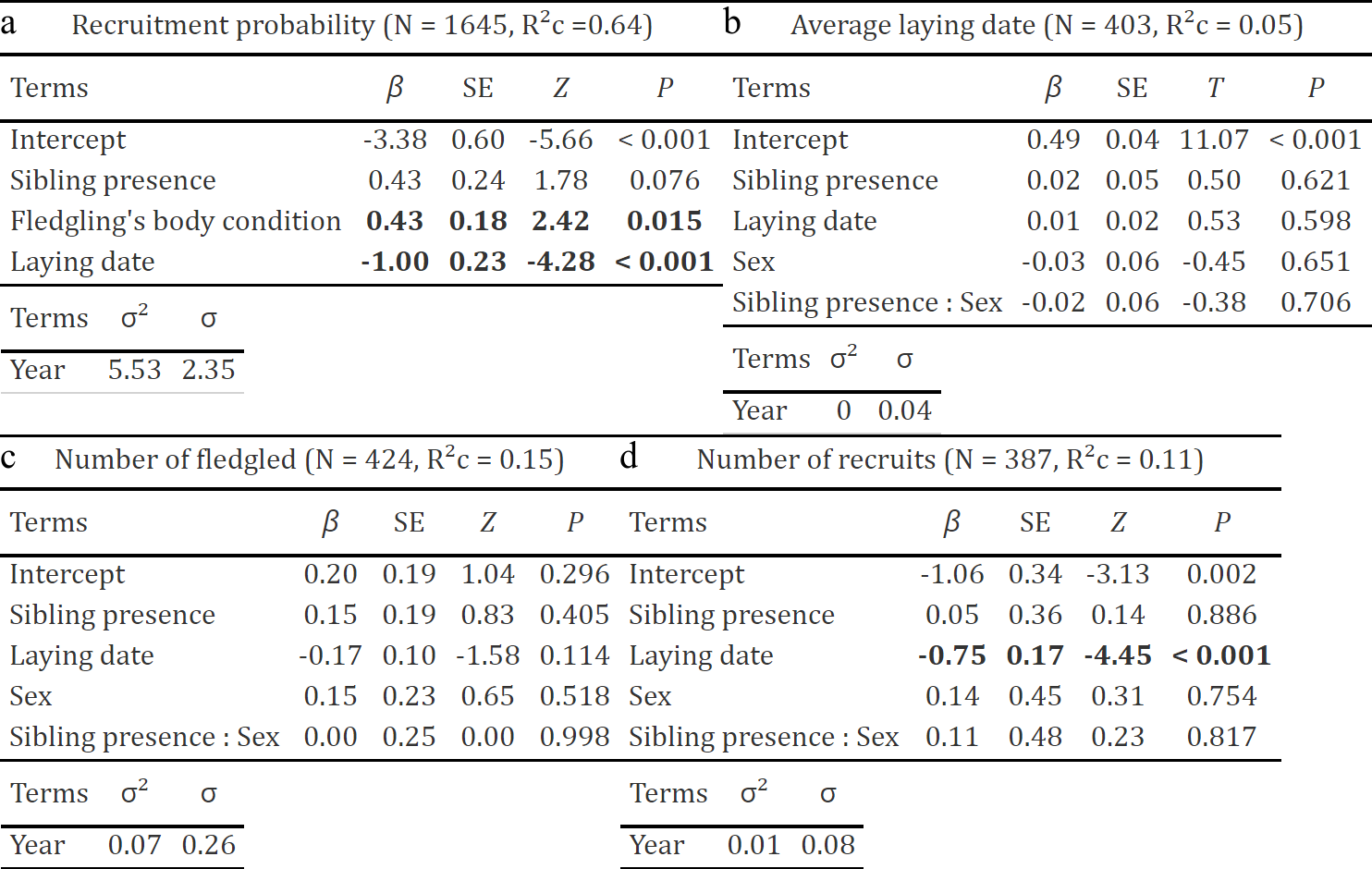

**Table S3**: Effect of sibling presence in core fledglings’ (a) and recruits’ (b, c, d) performance during their first six years of life in the recruitment probability (a), the timing of egg-laying (b) and the number of fledglings (c) and recruits produced (d). Inconsistencies in sample sizes between b and c are due to the exclusion of breeders whose laying date was unknown. Interaction effect between sex and sibling presence was dropped as it did not improve the model fit. Statistically significant terms are in bold.

## Effect of sibling’s cohabitation time on core fledglings from the control group

To test the effect of sibling cohabitation time on core fledglings’ body condition, recruitment probability and reproductive parameters (average laying date, numbers of fledglings and recruits produced) during the first six years after fledging, we rerun the five “facilitation” analyses. However, this time we only used core chicks from the control group (core fledglings that shared the nest with their junior sibling for at least six days), and the sibling presence variable was replaced by the siblings’ cohabitation time (number of shared days by the core offspring with its junior chick).


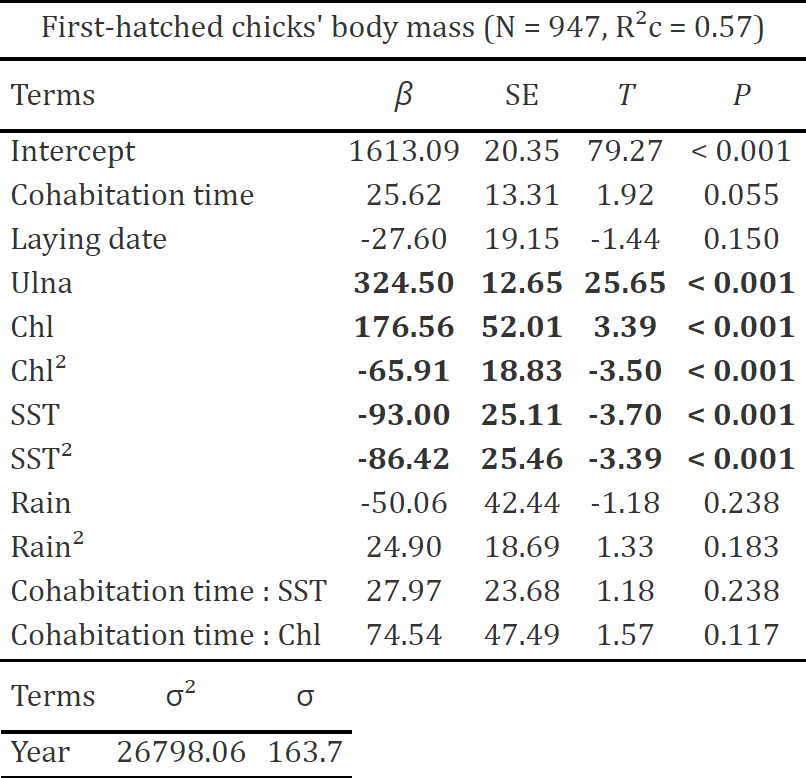


**Table S4**: Effect of siblings’ cohabitation time on core chick’s body mass at 70 days in broods of 2 chicks. Interaction effect between SST and siblings’ cohabitation time and between Chl-a and siblings’ cohabitation time on core chick’s body mass were dropped as they did not improve model fit. Statistically significant terms are in bold.


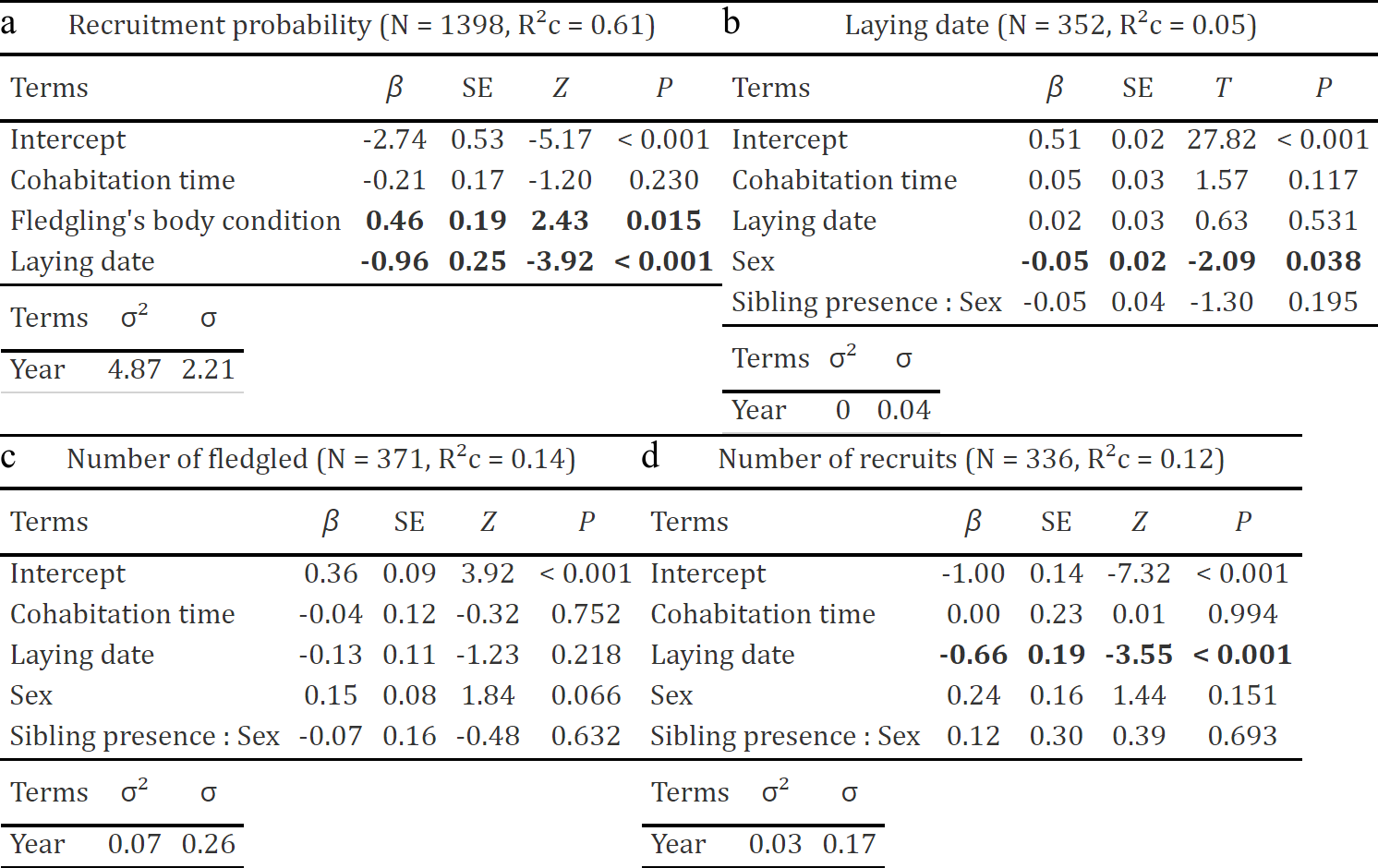


**Table S5**: Effect of sibling’s cohabitation time on core fledglings’ (a) and recruits’ (b, c, d) performance during their first six years of life at recruitment probability (a), timing of egg-laying (b) and number of fledglings (c) and recruits produced (d). Inconsistencies in sample sizes between b and c are due to the exclusion of individuals missing laying date. Interaction effect between sex and siblings’ cohabitation time was dropped as it did not improve model fit. Statistically significant terms are in bold.
