# Supplementary figures and images for: Parental overproduction allows siblicidal bird to adjust brood size to climate-driven prey variation
Source: Behav Ecol. 2024 Feb 1;35(2):arae007. doi: 10.1093/beheco/arae007 (PMC10878367; doi:10.1093/beheco/arae007)

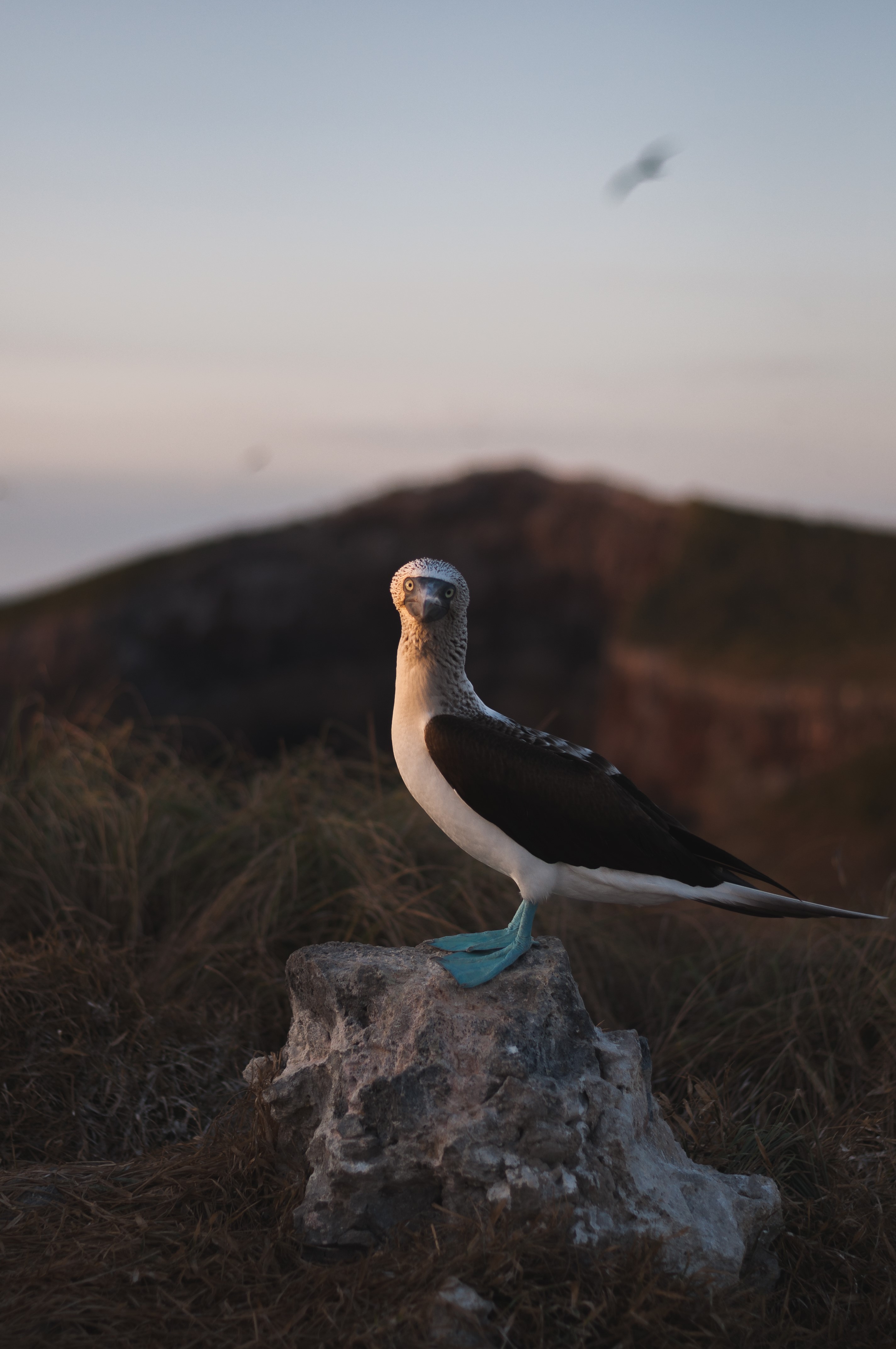

Supplement: arae007_suppl_Supplementary_Figure [file arae007_suppl_supplementary_figure.jpeg]
